# Mediterranean Diet and Parkinson’s Disease

**DOI:** 10.3390/ijms24010042

**Published:** 2022-12-20

**Authors:** Marco Bisaglia

**Affiliations:** 1 Department of Biology, University of Padova, Via Ugo Bassi 58/B, 35131 Padova, Italy; marco.bisaglia@unipd.it; 2 Study Center for Neurodegeneration (CESNE), 35100 Padova, Italy

**Keywords:** Parkinson’s disease, Mediterranean diet, gut-brain axis, resveratrol, olive oil

## Abstract

Parkinson’s disease (PD) is an age-related neurodegenerative disorder, diagnosed on the basis of typical motor disturbances, but also characterized by the presence of non-motor symptoms, such as rapid eye movement (REM)-sleep behavior disorders, olfactory impairment, and constipation, which are often prodromal to the onset of the disease. PD is often associated with the presence of oxidative brain injury and chronic neuroinflammation, with infiltration and accumulation of peripheral immune cells that have been found in affected brain regions of PD patients. Recently, the role of the gut-brain axis in the pathogenesis of PD is getting more and more attention, and several pieces of evidence indicate alterations in the gut microbiota of PD-affected patients. Diet exerts a central role in defining the microbiota composition and different dietetic patterns can result in a higher or lower abundance of specific bacteria that, in turn, can affect gut permeability and express anti- or pro-inflammatory metabolites. In the present review, the effects of the Mediterranean diet in modulating both PD onset and its progression will be considered with a special focus on the antioxidant and anti-inflammatory properties of this dietetic regimen as well as on its effects on the microbiota composition.

## 1. Introduction

Parkinson’s disease (PD) is a neurodegenerative disorder characterized by muscular rigidity, slowness of movements, postural instability, and resting tremors. These features are the result of the preferential degeneration of dopaminergic neurons in the substantia nigra pars compacta, which represents one of the pathological hallmarks of the disease. Other clinical manifestations include non-motor symptoms, such as depression, REMsleep behavior disorders, autonomic dysfunction, olfactory impairment, and constipation [1]. Interestingly these features often precede the onset of motor symptoms, so they are considered prodromal markers of the disease. Gastrointestinal (GI) dysfunctions, in particular, have been frequently described in PD patients. Usually, they appear at the premotor stages of the disease and tend to get worse as the disease progresses [1]. PD is mostly a sporadic disorder, but around 5–10% of all cases have a genetic origin [1]. Among the proteins associated with the familial forms of the disorder, α-synuclein (α-syn) has a prominent position. The protein has not only been the first to be linked to genetic forms of PD [2], but it is also one of the main components of the cytosolic inclusions referred to as Lewy bodies and Lewy neurites, which represent the second pathological hallmark of the disorder [3].

PD is currently considered a multifactorial disorder in which genetic susceptibilities and environmental factors contribute to the onset of the disease [4,5]. While the etiology of PD is not completely understood, compelling evidence supports the involvement of oxidative injury in the pathogenesis of the disease [6,7]. Due to its high energy demand, associated with high metabolic rate and oxygen consumption, the brain is particularly vulnerable to oxidative damage. Moreover, low levels of the antioxidant molecule glutathione have been found in the substantia nigra, in comparison to other brain regions [8], while the specific presence of the neurotransmitter dopamine inside dopaminergic neurons has been described to make this neuronal population more prone to oxidative injury [6]. Accordingly, high levels of oxidative modifications in DNA, proteins, and lipids, and a low amount of glutathione have been observed in post-mortem analyses of PD brains [6,7]. Of note, multiple pathological factors associated with the disease, such as mitochondrial dysfunctions and neuroinflammation, seem to promote the rising of oxidative damage. Mitochondria are commonly recognized as one of the main sources of intracellular ROS production, a process that is exacerbated in dysfunctional mitochondria when the electron transport chain is impaired [6]. In addition, during chronic activation of the neuroinflammatory response, the nicotinamide adenine dinucleotide phosphate (NADPH) oxidase subunit triggers the formation of superoxide anions and hydrogen peroxide while the inducible nitric oxide synthase produces high levels of nitric oxide, altogether leading to the formation of peroxynitrite radicals and nitrative stress [6]. Consistent with the involvement of mitochondrial dysfunction and neuroinflammation in PD pathogenesis, besides the presence of oxidative markers, post-mortem analyses also revealed impaired activity of mitochondrial complex I, as well as the presence of reactive microglia [6]. In addition, infiltration and accumulation of peripheral immune cells have been also found in affected brain regions of PD patients [9] The role of mitochondrial impairment and neuroinflammation in PD and reactive oxygen species (ROS) production is further supported by the finding that the PD-associated proteins parkin, PINK1, DJ-1, α-syn, and LRRK2 are involved in mitochondrial functioning and/or contribute to or modulate microglia activation [6].

One of the mechanisms through which alterations in redox homeostasis could lead to neuronal degeneration is linked to the subsequent impairment in protein homeostasis, a process that relies on several protein quality control steps, in a network of well-regulated processes ranging from protein synthesis to degradation. This proteostasis network is particularly important in the case of long-lived, post-mitotic cells like neurons and its failure seems to be implicated in the pathogenesis of several neurodegenerative disorders, including PD [10,11,12]. In this frame, oxidative injury has been described to impair proteostasis, by contributing to protein misfolding, in many cases mimicking the effect of the genetic mutations associated with the disease, and affecting both the ubiquitin proteasomal system and the autophagy-lysosomal system [10,11,12]. Consistent with alterations in the proteostasis network, PD is characterized by the accumulation of pathological oligomers and fibrils composed of misfolded α-syn, and several data indicate that the spreading of α-syn aggregates through a prion-like mechanism plays a crucial role in the progression of PD pathology. Several mechanisms have been described to mediate cell-to-cell transmission across diverse brain regions, including tunneling nanotubes, exosomes, and secretion of free pathological α-syn aggregates [13].

Interestingly, based on autopsy observations, Braak and co-workers proposed the hypothesis that the primary events of α-syn aggregation and spreading originate outside the brain, in the anterior olfactory nucleus and the dorsal motor nucleus of the vagus, further propagating through the cranial nerves from the peripheral nervous system to the brain and the other vulnerable areas in the central nervous system (CNS), including the striatum and substantia nigra [14]. Subsequent observations supported the gut-brain hypothesis, and α-syn aggregates have been described to be largely present in the gastrointestinal (GI) tract and enteric nervous system (ENS) of PD patients. Moreover., phosphorylated α-syn has been also reported in GI tissues from prodromal PD patients suggesting an involvement in the first asymptomatic phases of the disease [15]. However, contrasting data were also presented, and further work is required to draw definite conclusions as to whether gut-to-brain propagation of α-syn is a key causative step in PD [16,17]. Regardless of the direct (causal) involvement of α-syn spreading from the gut to the brain in the pathogenesis of PD, a growing body of evidence now indicates the presence of alterations in the microbiota of PD patients.

## 2. Gut Alterations in Parkinson’s Disease

A strict bidirectional connection exists between the brain and the GI tract. In fact, the brain controls the physiological functions of the GI tract with the sympathetic and parasympathetic autonomous divisions that connect the CNS with the gut, through the enteric nervous system (ENS). Although the ENS is able to operate independently of the CNS, it may be influenced by it through the vagus nerve and prevertebral ganglia [18].

The human GI tract accommodates trillions of microorganisms such as bacteria, viruses, and fungi, and a great variety of microbes inhabit the human gut. The gut microbiota is mostly constituted by phyla Firmicutes and Bacteroidetes, with other phyla including Proteobacteria, Actinobacteria, Fusobacteria, Verrucomicrobia, Bifidobacteria, Lentisphaerae and Spirochaetes that represent around 1% of microbes [18]. All these microorganisms play essential functions since they participate in food digestion and the following absorption of nutrients in the GI tract, and in giving protection against pathogens. Moreover, they modulate GI functions by contributing, for instance, to the mucosal immune function, intestinal permeability, visceral pain, stress responses, and ENS activity [18].

Due to the important role of gut microbiota in human health, many studies were performed to evaluate possible differences in intestinal microbiota composition between PD patients and healthy controls. Overall, these analyses focused on the bacterial composition of the human gut, while neglecting both viruses and fungi, and found significant alterations in the microbiota of PD patients, regardless of sequence technique and bioinformatics methodologies used. As summarized in recent reviews [15,17,18,19,20], some of the notable alterations in gut microbial composition in PD include, at the family level, an increase in the relative abundance of Bifidobacteriaceae, Christensenellaceae, Enterobacteriaceae, Enterococcaceae, Lactobacillaceae, Ruminococcaceae, and Verrucomicrobiaceae, and a decrease in the relative abundance of Prevotellaceae and Lachnospiraceae. Other consistent features at the genus level include an increased abundance of *Akkermansia*, *Bifidobacterium,* and *Lactobacillus*, and a decreased abundance of *Blautia*, *Faecalibacterium*, *Prevotella,* and *Roseburia*.

The nature of the microorganisms whose abundance is altered in PD patients can shed some light on the mechanisms that might correlate gut dysbiosis with PD pathogenesis. Although changes in the microbiota population can promote PD through different pathways, including the modulation of dopamine activity and bioavailability [20,21], two appear particularly relevant. The former relies on the contribution of the microbiota to gut permeability, the latter on its participation in the inflammatory response. In fact, the intestinal epithelial barrier prevents the passage of pro-inflammatory molecules from the intestinal lumen to systemic circulation, and the gut microbiota plays a critical role as a regulator of intestinal barrier integrity.

**Figure 1:**
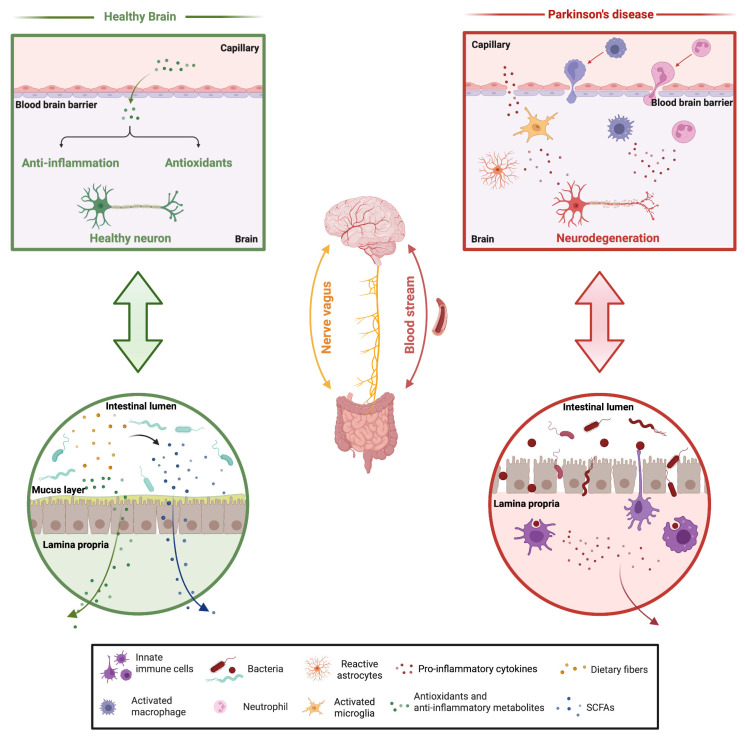
Proposed contribution of gut dysbiosis to PD. In healthy individuals, the gut microorganisms participate in the formation of the intestinal mucosal layers through the synthesis of SCFAs and regulate the host immune response. They also participate in the production of antioxidant and anti-inflammatory metabolites that can reach the brain where they exert neuroprotection. In PD patients, alterations in the gut microbiota population affect the production of SCFAs leading to gut permeability. At the same time, pro-inflammatory cytokines are produced that enhance the systemic inflammatory response. Long-term activation of peripheral immune cells promotes the breakage of the blood-brain barrier, leading to chronic neuroinflammation (created with BioRender.com, accessed on 12 October 2022).

*Blautia*, *Faecalibacterium*, and *Roseburia*, as well as Lachnospiraceae family and Prevotellaceae members, which are all less abundant in PD patients, participate in the formation of the intestinal mucosal layer and the production of short-chain fatty acids (SCFAs) [15,17,18,19]. For instance, *Prevotella* species play an important role in the synthesis of mucus proteins called mucins, which are the principal structural and functional components of gastric mucus, and its depletion can contribute to increased gut permeability. Consistent with the discovery of a reduced abundance of SCFA-producing bacteria in the gut microbiota, data from fecal metabolomics platforms have shown a reduction in levels of the fecal SCFAs butyrate, acetate, and propionate in PD patients in comparison to healthy controls [17,19]. SCFAs, which are produced through the fermentation of dietary fibers in the gut, exert a crucial function on the integrity of the intestinal barrier. As a consequence, reduced levels of the SCFA-producing bacteria might affect gut permeability (Figure 1). SCFAs and, in particular, butyrate are also thought to play an anti-inflammatory role through modulation of the nuclear factor kappa B (NF-κB) transcription factor. In addition to this cellular pathway, these bacterial populations have also been described to promote the expression of anti-inflammatory cytokines while suppressing the expression of pro-inflammatory ones [19]. Conversely, members of the gram-negative Enterobacteriaceae family, whose relative abundance is increased in PD patients, are considered to foster gut inflammation through the production of the endotoxins lipopolysaccharide (LPS) [19], which stimulate the release of pro-inflammatory cytokines that, in turn, may produce an inflammatory response in the systemic circulation and the brain (Figure 1) [15,19].

Consistent with this proposed pathological mechanism, PD patients are characterized by both peripheral and CNS inflammation together with increased intestinal permeability [22,23]. In fact, increased levels of pro-inflammatory cytokines, such as TNF-α, IL-1α and IL-1β, and fecal markers of inflammation have been found in colonic biopsies and stool of PD patients, together with higher gut permeability and submucosal invasion of *Escherichia coli* and α-syn aggregation [24]. Interestingly, Toll-like receptor 4 (TLR4)-mediated inflammatory signaling could be responsible for the increased gut permeability, since a higher number of TLR4 and CD3+ T cells, as well as a lower number of tight junction-related proteins, have been found in mucosal biopsies of people with PD with respect to healthy controls [24]. In addition, gut dysbiosis could promote the trans vagal spread of α-syn aggregates to the dorsal motor neurons of the vagus and into the brain [24].

In conclusion, the picture that emerges is that, due to the activation of the intestinal inflammatory response and the gut leakage, the peripheral activated immune cells can migrate into the brain, promoting the breakage of the blood-brain barrier and connecting in such a way the systemic inflammation to the brain [23,25,26].

The enhanced relative abundance of *Akkermansia*, *Bifidobacterium*, and *Lactobacillus* in PD patients is more complex to rationalize as they are usually considered protective bacteria and are often found in “probiotic” preparations. It has been suggested that their increase could depend on their high adaptability to survive in an altered gut environment [23]. The principal differences in terms of microbiota composition and its downstream effects are summarized in Table 1.

## 3. Mediterranean Diet and Parkinson’s Disease

Since no therapeutic treatments exist to stop the progression of PD, preventive actions might be adopted to minimize the risk factors and reduce the probability of developing the disease. In this frame, nutrition could represent an environmental factor able to promote or prevent the onset of PD, as is the case of other pathological conditions. For instance, the Western diet, characterized by a high caloric intake of energy-dense foods, with high levels of processed and fried foods, red meat, refined sugars, and saturated fatty acids, has been linked to increased prevalence and severity of numerous disorders including cardiovascular diseases and type2 diabetes [27]. Interestingly, from a systematic analysis of the Global Burden of PD between 1990 and 2016, a positive trend emerged between the socio-demographic index (SDI) of a country and the age-standardized prevalence of PD as well as the age-standardized rate of disability-adjusted life-years, defined as the sum of years lived with disability and years of life lost [28]. Although the reason for this association is not clear and cannot be easily rationalized, one could speculate that this could be partially due to the large diffusion of the Western diet in high SDI countries and, in particular, in high-income North America.

**Figure 2:**
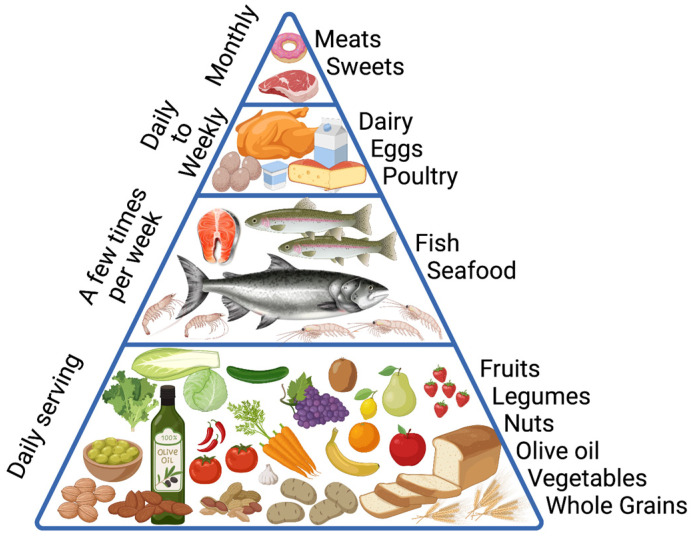
Mediterranean diet pyramid, adapted from [33]. According to the eating habits of long-living adults in the Mediterranean basin, the pyramid summarizes the type and frequency of foods that constitute the Mediterranean dietetic pattern. The pyramid is divided into daily, weekly, and monthly frequencies (created with BioRender.com, accessed on 12 October 2022).

Conversely, the Mediterranean diet (MeDiet), which traditionally represents the nutritional pattern of the Mediterranean basin (Greece, Spain, and southern regions of Italy), is widely considered to beneficially impact health and longevity [29]. The MeDiet is characterized by a high intake of fresh vegetables and fruits, whole grain cereals, legumes, seeds, and nuts, together with consistent use of olive oil, by the moderate consumption of milk, cheese, yogurt, potatoes, eggs, fish, poultry, and red wine, and by low amounts of red meat and saturated fats (Figure 2). Collectively, the MeDiet is rich in antioxidants, anti-inflammatory agents, minerals, and vitamins [30,31]. If the big difference between the Western diet and MeDiet is the high fat and processed red meat intake of the former and the large amount and variety of plant-based foods of the latter, which also includes low to moderate consumption of meat, dairy products, eggs, and fish, it is worth mentioning that, as recently reviewed [32] other dietary patterns share similar characteristic with the MeDiet. For instance, Vegan and Vegetarian diets are based on a wide amount of plant foods, with the former excluding any animal food product, while the latter includes seafood, dairy products, and eggs in addition to vegetables, fruits, whole grains, legumes, nuts, and seeds. A more detailed comparison of food intake among the aforementioned dietary patterns can be found elsewhere [32] and is beyond the scope of the present review, which will focus on the MeDiet for which most information is available. While further research is warranted to study the effects of other plant-based dietary patterns, some of the results presented in this review could be cautiously translated to other MeDiet-related and/or Plant-based diets.

Although the effect of the MeDiet on PD has not been exhaustively investigated and the results are sometimes contradictory, the overall emerging picture is that the MeDiet is somehow protective against PD onset and progression. A first prospective study was carried out on two large American cohorts of men and women: participants in the Health Professionals Follow-Up Study (HPFS) and the Nurses’ Health Study (NHS). After 16 years of follow-up, dietary patterns characterized by a high intake of fruit, vegetables, legumes, whole grains, nuts, fish, and poultry and a low intake of saturated fat, and a moderate intake of alcohol, were associated with a reduced risk of PD [34]. Similar results, with some distinctions, were reported in two other longitudinal studies. In the first, based on a large cohort of Swedish women, a higher adherence to the MeDiet in middle age was associated with a lower risk for PD [35]. The second, in addition to the canonical MeDiet, took into consideration an improved diet pattern called MIND (Mediterranean-DASH Diet Intervention for Neurodegenerative Delay), which is a combination of the MeDiet and the DASH (Dietary Approaches to Stop Hypertension) diet [36]. Although most food groups are similar or identical to those found in the MeDiet, the MIND diet uniquely rewards leafy green, berry, and poultry intake while minimizing the consumption of fried food and sweets. Milk, potato, and fruit intake are also discarded [27]. In models adjusted for age, sex, smoking, total energy intake, body mass index, and depressive symptoms, higher MIND diet scores were associated with a decreased risk of parkinsonism and a slower rate of parkinsonism progression. More moderate protective associations were observed for the Mediterranean diet but not for the DASH diet [37]. A similar picture emerges from other observational studies. In a small case-control study, based on approximately 250 PD patients and 200 healthy controls, higher MeDiet adherence was associated with lower odds for PD and lower MeDiet scores were associated with earlier PD age at onset [38]. In another cross-sectional study, higher adherence to the MIND diet was significantly associated with a higher age at disease onset, especially in women, with the sex-dependent differences attributed to females being more adherent to the diet throughout the course [27]. In the same study, the effects of two slightly different MeDiet patterns were still significant, although lower than MIND [27]. Interestingly, another cross-sectional analysis, based on more than 1000 individuals with self-reported idiopathic PD, provided evidence that targeted nutrition could also affect the progression of the disease. More specifically, most of the foods associated with a reduced rate of PD progression are representative of the MeDiet, such as fresh vegetables, fresh fruit, nuts and seeds, nonfried fish, olive oil, wine, fresh herbs, and spices; while foods associated with more rapid PD progression include canned fruits and vegetables, fried foods, beef, ice cream, yogurt, and cheese [39]. Consistent with this study, MeDiet administration for 10 weeks in Iranian patients with idiopathic PD augmented the total score of cognitive assessment, with marked improvements in executive function, language, attention, concentration, and active memory [40], and showed benefits in the locomotor performance [41]. Since constipation represents one of the most common nonmotor symptoms that often precede PD diagnosis, the results described in two prospective studies about the effects of the MeDiet on the prodromal signs of PD appear particularly relevant. In a Greek large-scale, population-based study based on more than 1700 PD-free individuals aged more than 65 years, the median probability of prodromal PD was described to be around 2%. Interestingly, a lower probability for prodromal PD in the higher MeDiet adherence groups was noted, driven mostly by nonmotor markers of prodromal PD, depression, constipation, urinary dysfunction, and daytime somnolence. Compared to participants in the lowest quartile of MeDiet adherence, those in the highest quartile were associated with a ~20% lower probability of prodromal PD [42]. Accordingly, in another longitudinal study based on the two large, previously mentioned HPFS and NHS cohorts, increased long-term MeDiet adherence was inversely associated with constipation, excessive daytime sleepiness, and depressive symptoms [43]. Since the prodromal features investigated do not necessarily mean an individual will eventually develop clinically manifest PD, future follow-up investigations on these cohorts will be welcome. In this frame, two recent studies evaluated the effects of short-term MeDiet adherence on gastrointestinal dysfunctions in PD patients. Both works observed improvement in the constipation clinical symptoms, without observing markers of intestinal permeability or inflammation [22,44]. However, while no difference in the fecal microbial diversity was observed in one of the studies [22], the other detected an increased proportion of *Roseburia* at the end of the treatment [44]. As aforementioned, the participation of *Roseburia* in the formation of the intestinal mucosal layer and the production of SCFAs could account for the observed improved constipation.

The different clinical trials evaluating the effect of MeDiet on PD onset and progression are summarized in Table 2.

## 4. Antioxidant and Anti-Inflammatory Properties of the Mediterranean Diet

One of the main beneficial properties of the MeDiet is its generally recognized capability to positively affect both the redox unbalance and inflammatory response. The first indication came from a study performed long ago, based on about 10,000 middle-aged men from 14 cohorts in 7 countries, in which it was observed that the MeDiet pattern was associated with a low incidence of coronary heart disease and results beneficial in a number of other chronic diseases [45]. Since then, several additional studies provided indications of the effects of the MeDiet in reducing the incidence of inflammatory disease states [46]. As neuroinflammation and oxidative stress are recognized factors involved in PD, the beneficial effects of the MeDiet described in the previous section could rely on the antioxidant and anti-inflammatory properties of this dietary pattern. Among the different elements found in the MeDiet, vitamins, omega-3 polyunsaturated fatty acids (ω3-PUFAs) and polyphenols have been largely studied for their protective properties. Vitamins and carotenoids are among the main constituents of fruits and vegetables and possess antioxidant properties that could be helpful to contrast redox unbalance, reducing the risk of PD. Nevertheless, at present, there is no indication that their intake is protective against PD risk [19,47]. Consumption of PUFAs is also an element of the MeDiet and three principal types of ω3-PUFAs exist which include eicosapentaenoic acid (EPA), docosahexaenoic acid (DHA, typically from fish oil), and alpha-linolenic acid (ALA, typically from plant sources) [15,30]. Ω3-PUFAs are important constituents of the membrane phospholipids and play an important role in modulating the chemical-physical properties of membranes, which in turn influence cellular functions. There are many ways through which ω3-PUFAs may impact the brain and be beneficial in the prevention and/or treatment of PD, including their antioxidant and anti-inflammatory properties, and different mechanisms of action behind their anti-inflammatory activity have been proposed [15,19,30]. Polyphenols are a big and heterogeneous group of compounds, structurally characterized by the presence of one or more aromatic rings carrying one or more hydroxyl groups and bounded to different structural units. Polyphenols are the main antioxidant molecules of our diet, found in vegetables, such as broccoli, cabbage, and onion, extra-virgin olive oil, fruit, such as grapes, apples, pears, and cherries, legumes, cereals, and chocolate, as well as in drinks such as red wine, coffee, and tea [30,31,48]. Among the different polyphenols, resveratrol and the olive tree derivatives oleuropein and hydroxytyrosol are among the most studied.

### 4.1. Resveratrol

Resveratrol (3,5,4′-trihydroxy-trans-stilbene) is a polyphenolic compound belonging to the stilbene family, which is found in several plants and trees, including *Arachis hypogaea* (peanut), *Vitis vinifera* (grape wine), *Pinus*, and *Eucalyptus*. Its intake through the diet is mainly related to the assumption of peanuts, blueberries, and cranberries with grape skins and red wine that constitute the primary sources in the human diet [31,49].

Resveratrol is a low molecular weight molecule composed of two phenol rings, linked together by a double styrene bond, and by three hydroxyl groups. These functional groups have been suggested to be essential for resveratrol antioxidant activity being responsible for the free radical scavenging activity and metal chelation [31].

Approximately 70% of the ingested resveratrol is absorbed at the intestinal level through passive diffusion or following the formation of complexes with membrane transporters. The non-absorbed portion reaches the colon where it is degraded by gut microbiota leading to low molecular weight compounds. Once absorbed, resveratrol is readily metabolized at the hepatic level to form the corresponding glucuronide or sulfate derivatives [31,49]. These compounds, as well as small amounts of unmodified resveratrol, are found in the bloodstream and are excreted in urine and feces. Resveratrol and its metabolites have been found distributed in several organs such as the liver, kidney, and lungs. Importantly, resveratrol and its metabolites are also able to cross the blood-brain barrier, as they have been detected in the cerebrospinal fluid [31,49].

Many protective functions have been ascribed to resveratrol, including antioxidant, anti-inflammation, and neuroprotective activity [31]. Acting as a free radical scavenger, it counteracts excessive reactive oxygen species production and lipid peroxidation and modulates inflammation. It is also able to reduce inflammation by inhibiting the NF-κB inflammation pathways and promoting the nuclear translocation of the Nuclear Factor Erythroid 2-Related Factor 2 (Nrf2), which activates the transcription of antioxidant enzymes. Moreover, resveratrol has been described to promote mitochondrial biogenesis and activity through the adenosine monophosphate-activated protein kinase (AMPK) and PGC-1α expression [31,48].

### 4.2. Olive Tree-Derived Polyphenols

One of the main characteristics of the MeDiet is the daily consumption of olive oil with the consequence that olive oil polyphenols could in part be responsible for the beneficial effect of this dietary pattern. The olive tree (*Olea europaea*) synthesizes several polyphenols, especially in leaves and drupes, which are used as a defense mechanism against microbes and fungi. Curiously, leaves contain a higher concentration of total polyphenols than olive fruit [31,50]. While tyrosol, oleuropein, and hydroxytyrosol are the main polyphenols present in black and green olives, because of the oil production procedures and aging processes that transform oleuropein into hydroxytyrosol, the latter represents the main phenolic compound found in olive oil with a median content ranging from 50 to 200 mg/kg. This concentration is affected by several factors such as the geographical area, age of the trees, degree of ripeness, extraction methodology, and storage conditions [31,49,50]. Both the composition and concentration of polyphenols in olive oil, together with the degree of their absorption, metabolism, and bioavailability are key determinants of the beneficial effects associated with olive oil.

After ingestion, once in the stomach, oleuropein is usually hydrolyzed into hydroxytyrosol, although a small unmodified fraction enters the small intestine, where it can be either absorbed or it can reach the colon, generating more diverse microbial metabolites. Hydroxytyrosol and tyrosol are highly absorbed in a 40–95% range. Interestingly, increased bioavailability of these compounds was described when they were administered as an olive oil solution compared to an aqueous solution, highlighting the importance of the vehicle in determining their bioavailability [46,49,50,51]. After their absorption, hydroxytyrosol, and tyrosol are rapidly metabolized into the corresponding glucuronide and sulfate derivatives, which are then excreted in the urine. Hydroxytyrosol, tyrosol, and their metabolites have been found widely distributed in several organs and tissues such as skeletal muscles, the liver, heart, kidney, and lungs. Very importantly, these molecules, as well as oleuropein, have also been found in the brain demonstrating their capability to cross the blood-brain barrier, a condition sine qua non to exert neuroprotective effects [31,49,50].

As aforementioned, the protective effects associated with olive oil consumption seem to be mostly related to the antioxidant and anti-inflammatory activity of its polyphenolic fractions. First, olive oil-derived polyphenols are reactive oxygen species scavengers and as such, they can increase cellular endogenous antioxidant defenses. Moreover, several studies have shown that hydroxytyrosol, tyrosol, and oleuropein can activate the Nrf-2 signaling pathway inducing a cellular defense response against oxidative damage and pro-inflammatory stimuli. In addition to suppressing the NF-κB-dependent inflammatory response through the activation of the Nrf-2 pathway, hydroxytyrosol and tyrosol have also been described as direct modulators of the NF-κB pro-inflammatory pathway, by acting on upstream kinases, and this activity is shared by their glucuronide and sulfate derivatives [51]. Finally, oleocanthal, another natural phenolic compound found in olive oil, has been shown to act as a natural anti-inflammatory compound by inhibiting the cyclooxygenase COX-2 activity in the prostaglandin-biosynthesis pathway, with an efficacy comparable to that of the classical commercially available inhibitors [31,51]. An exhaustive description of antioxidant and anti-inflammatory protective properties of olive polyphenols, including their mechanisms of action, has been recently published ([52] and is beyond the scope of this review.

## 5. Mediterranean Diet Influences the Gut Microbiota Composition

In addition to the existing evidence on the impact of the MeDiet on the antioxidant and anti-inflammatory response, an alternative and non-exclusive protective pathway could be related to the effects mediated by this dietary pattern at the intestinal level. The GI tract is one of the largest interfaces between the host and the environment and the gut microbiota consists of a complex ecological community. As previously highlighted, the gut microbiota plays several beneficial functions for the host such as the maintenance of the integrity of the intestinal epithelium, absorption and synthesis of nutrients and their metabolites, protection against pathogens, and regulation of host immunity response [19]. The composition of the gut microbiota is established at birth and continues to evolve until adult life when it becomes relatively stable [53]. Antibiotic intake, infections, and more in general, environmental and stress factors can influence, alter, and modify gut microbiota composition, and dysbiosis has been linked to several disorders [54]. Among the lifestyle factors, diet robustly impacts the intestinal microbiota. For instance, the Western diet, which is characterized by high consumption of proteins from animal sources, promotes the growth of LPS-containing bacteria and reduces the abundance of SCFA-producing bacteria, possibly leading to systemic inflammation and damage to the blood-brain barrier [15,20,23]. Interestingly, such a situation has also been described in PD patients. In this frame, a possibility exists that the protection postulated for the MeDiet against PD onset and progression is related to the modulation of the composition of the gut microbiota mediated by this dietary pattern. In fact, the MeDiet is rich in fibers and their consumption may promote the growth of polysaccharide-degrading bacteria, which use dietary fiber and polysaccharides to produce SFCAs while reducing LPS-containing bacteria [15,20,23]. Accordingly, high adherence to the MeDiet has been associated with increased microbial richness, upregulation of beneficial microbes, and enhanced SCFAs [55,56,57]. Of note, similar effects on gut microbiota have been also described in people following Vegetarian and Vegan diets [58], which, like the MeDiet, are particularly rich in fiber. The increased production of SCFAs, in turn, may fortify the intestinal barrier and inhibit inflammation.

## 6. Conclusions

Considering the increase in life expectancy of the world population, age-related neurodegenerative disorders, including PD, represent a big health challenge for the future. At present, PD is still an uncurable disorder, with only symptomatic treatments available so that preventive actions might be adopted to minimize the risk factors and reduce the probability of developing the disease. The gut-brain axis is getting increasing attention for its potential role in the pathogenesis of PD, opening the road for new therapeutic preventive strategies, and nutrition could represent an important aspect to take into consideration to reduce the probability or even prevent the onset of PD. Although the clinical trials carried out until now present several potential caveats and new well-designed long terms studies are necessary, the currently available data support a protective role of the MeDiet against PD onset and progression. The mechanism by which the MeDiet could confer health benefits against PD could depend on its anti-inflammatory and antioxidant properties, being rich in vitamins, ω3-PUFAs, and polyphenols. Emerging clinical and experimental evidence seems to suggest that the beneficial effects obtained with a MeDiet-based dietary regimen might be also mediated by the intestinal microbiota.

Given the findings summarized in the present review, people should be encouraged to eat a diet rich in fruit, fresh vegetables, whole grains, and olive oil while limiting their intake of red meat and sugar-rich foods. Although these dietary habits should be promoted from an early age since long-term MeDiet adherence has been shown to protect also from the prodromal features of PD that can manifest decades before the official diagnosis, it is never late to start a healthy diet since MeDiet adherence in PD patients was described to reduce the rate of disease progression. We should also reflect on the fact that, while the MeDiet has been shown to be protective in several studies carried out all over the world, a consequence of globalization is the possible loss of traditional dietary habits, including the MeDiet, in favor of the Western diet.

## Figures and Tables

**Table 1:** Most consistent microbiota alterations measured in PD patients with respect to healthy individuals (from [15,17,18,19,20]).

Microbiota Alterations in PD Patients	Downstream Metabolites	Local Impact in PD Patients	Impact on Brain in PD Patients
↑ *Akkermansia*	Mucin-degrading enzymes	Immune gut homeostasis	
↑ *Bifidobacterium,* ↑ *Lactobacillus*	Tight junction proteins	Infection in immune-compromised individuals	
↑ Enterobacteriaceae family	LPS	↑ Inflammation	↑ BBB permeability ↑ Neuroinflammation
↓ *Blautia* ↓ *Faecalibacterium* ↓ *Roseburia*	SCFAs	↓ Gut permeability (mucus formation) ↓ Inflammation (via the NF-κB pathway)	↑ BBB permeability ↓ Microglia-mediated protection
↓ Prevotellaceae family	Mucins synthesis	↓ Gut permeability (mucus formation)	↑ BBB permeability ↑ Neuroinflammation

**Table 2:** Clinical trials evaluating the effects of the MeDiet on PD.

Typology of the Study	Country	Period of Follow Up	Individuals Involved	Dietary Pattern	Results	Ref
Longitudinal	USA	16 years	49,692 + 81,676 (2 cohorts)	MeDiet-related	MeDiet-related diet protects against PD onset	[34]
Longitudinal	Sweden	20 years	47,128	MeDiet	MeDiet reduces PD risk	[35]
Longitudinal	USA	4.6 years	706	MIND; DASH; MeDiet	MIND reduces PD risk MIND and MeDiet slow PD progression	[37]
Case-control	USA		257 PD + 198 controls	MeDiet	MeDiet reduces PD risk	[38]
Cross-sectional	Canada		167 PD + 119 controls	MIND MeDiet	MIND and MeDiet protect against PD onset	[27]
Cross-sectional	USA		1053 PD	MeDiet-related	MeDiet-related foods slow PD progression	[39]
Randomized clinical trial	Iran	10 weeks	80 PD	MeDiet	MeDiet improves both cognitive functions and locomotor performance in PD patients	[40]
Cross-sectional	Greece		1731	MeDiet	MeDiet protects against prodromal PD symptoms	[42]
Longitudinal	USA	16 years	47,679	MeDiet-related	MeDiet-related protects against prodromal PD symptoms	[43]
Randomized clinical trial	USA	8 weeks	52 PD	MeDiet	MeDiet improves constipation symptoms in PD patients	[22]
Single-arm clinical trial	USA	5 weeks	8 PD + 8 controls	MeDiet	MeDiet improves constipation symptoms in PD patients	[44]

## Data Availability

Not applicable.

## References

[1] Karimi-Moghadam A., Charsouei S., Bell B., Jabalameli M.R. (2018). Parkinson Disease from Mendelian Forms to Genetic Susceptibility: New Molecular Insights into the Neurodegeneration Process. Cell. Mol. Neurobiol..

[2] Polymeropoulos M.H., Lavedan C., Leroy E., Ide S.E., Dehejia A., Dutra A., Pike B., Root H., Rubenstein J., Boyer R. (1997). Mutation in the Alpha-Synuclein Gene Identified in Families with Parkinson’s Disease. Science.

[3] Spillantini M.G., Schmidt M.L., Lee V.M.Y., Trojanowski J.Q., Jakes R., Goedert M. (1997). Alpha-Synuclein in Lewy Bodies. Nature.

[4] Cannon J.R., Greenamyre J.T. (2013). Gene-Environment Interactions in Parkinson’s Disease: Specific Evidence in Humans and Mammalian Models. Neurobiol. Dis..

[5] Kalia L.V., Lang A.E. (2015). Parkinson’s Disease. Lancet.

[6] de Lazzari F., Bubacco L., Whitworth A.J., Bisaglia M. (2018). Superoxide Radical Dismutation as New Therapeutic Strategy in Parkinson’s Disease. Aging Dis.

[7] Bisaglia M., Bubacco L. (2020). Copper Ions and Parkinson’s Disease: Why Is Homeostasis So Relevant?. Biomolecules.

[8] Li S., Thompson S.A., Woods J.S. (1996). Localization of γ-Glutamylcysteine Synthetase MRNA Expression in Mouse Brain Following Methylmercury Treatment Using Reverse Transcription in Situ PCR Amplification. Toxicol. Appl. Pharmacol..

[9] Galiano-Landeira J., Torra A., Vila M., Bové J. (2020). CD8 T Cell Nigral Infiltration Precedes Synucleinopathy in Early Stages of Parkinson’s Disease. Brain.

[10] Nakamura T., Oh C.K., Zhang X., Lipton S.A. (2021). Protein S-Nitrosylation and Oxidation Contribute to Protein Misfolding in Neurodegeneration. Free Radic. Biol. Med..

[11] van Dam L., Dansen T.B. (2020). Cross-Talk between Redox Signalling and Protein Aggregation. Biochem. Soc. Trans..

[12] Höhn A., Tramutola A., Cascella R. (2020). Proteostasis Failure in Neurodegenerative Diseases: Focus on Oxidative Stress. Oxid. Med. Cell. Longev..

[13] Streubel-Gallasch L., Giusti V., Sandre M., Tessari I., Plotegher N., Giusto E., Masato A., Iovino L., Battisti I., Arrigoni G. (2021). Parkinson’s Disease-Associated LRRK2 Interferes with Astrocyte-Mediated Alpha-Synuclein Clearance. Mol. Neurobiol..

[14] Braak H., del Tredici K., Rüb U., de Vos R.A.I., Jansen Steur E.N.H., Braak E. (2003). Staging of Brain Pathology Related to Sporadic Parkinson’s Disease. Neurobiol. Aging.

[15] Jackson A., Forsyth C.B., Shaikh M., Voigt R.M., Engen P.A., Ramirez V., Keshavarzian A. (2019). Diet in Parkinson’s Disease: Critical Role for the Microbiome. Front. Neurol..

[16] Lionnet A., Leclair-Visonneau L., Neunlist M., Murayama S., Takao M., Adler C.H., Derkinderen P., Beach T.G. (2018). Does Parkinson’s Disease Start in the Gut?. Acta Neuropathol..

[17] Tan A.H., Lim S.Y., Lang A.E. (2022). The Microbiome-Gut-Brain Axis in Parkinson Disease—From Basic Research to the Clinic. Nat. Rev. Neurol..

[18] Thangaleela S., Sivamaruthi B.S., Kesika P., Bharathi M., Chaiyasut C. (2022). Role of the Gut-Brain Axis, Gut Microbial Composition, Diet, and Probiotic Intervention in Parkinson’s Disease. Microorganisms.

[19] Lorente-picón M., Laguna A. (2021). New Avenues for Parkinson’s Disease Therapeutics: Disease-Modifying Strategies Based on the Gut Microbiota. Biomolecules.

[20] Hamamah S., Hajnal A., Covasa M. (2022). Impact of Nutrition, Microbiota Transplant and Weight Loss Surgery on Dopaminergic Alterations in Parkinson’s Disease and Obesity. Int. J. Mol. Sci..

[21] Hamamah S., Aghazarian A., Nazaryan A., Hajnal A., Covasa M. (2022). Role of Microbiota-Gut-Brain Axis in Regulating Dopaminergic Signaling. Biomedicines.

[22] Rusch C., Beke M., Tucciarone L., Dixon K., Nieves C., Mai V., Stiep T., Tholanikunnel T., Ramirez-Zamora A., Hess C.W. (2021). Effect of a Mediterranean Diet Intervention on Gastrointestinal Function in Parkinson’s Disease (the MEDI-PD Study): Study Protocol for a Randomised Controlled Trial. BMJ Open.

[23] Sun X., Xue L., Wang Z., Xie A. (2022). Update to the Treatment of Parkinson’s Disease Based on the Gut-Brain Axis Mechanism. Front. Neurosci..

[24] Brown E.G., Goldman S.M. (2020). Modulation of the Microbiome in Parkinson’s Disease: Diet, Drug, Stool Transplant, and Beyond. Neurotherapeutics.

[25] Harms A.S., Thome A.D., Yan Z., Schonhoff A.M., Williams G.P., Li X., Liu Y., Qin H., Benveniste E.N., Standaert D.G. (2018). Peripheral Monocyte Entry Is Required for Alpha-Synuclein Induced Inflammation and Neurodegeneration in a Model of Parkinson Disease. Exp. Neurol..

[26] Mou Y., Du Y., Zhou L., Yue J., Hu X., Liu Y., Chen S., Lin X., Zhang G., Xiao H. (2022). Gut Microbiota Interact With the Brain Through Systemic Chronic Inflammation: Implications on Neuroinflammation, Neurodegeneration, and Aging. Front. Immunol..

[27] Metcalfe-Roach A., Yu A.C., Golz E., Cirstea M., Sundvick K., Kliger D., Foulger L.H., Mackenzie M., Finlay B.B., Appel-Cresswell S. (2021). MIND and Mediterranean Diets Associated with Later Onset of Parkinson’s Disease. Mov. Disord..

[28] Ray Dorsey E., Elbaz A., Nichols E., Abd-Allah F., Abdelalim A., Adsuar J.C., Ansha M.G., Brayne C., Choi J.Y.J., Collado-Mateo D. (2018). Global, Regional, and National Burden of Parkinson’s Disease, 1990-2016: A Systematic Analysis for the Global Burden of Disease Study 2016. Lancet Neurol..

[29] Mazza E., Ferro Y., Pujia R., Mare R., Maurotti S., Montalcini T., Pujia A. (2021). Mediterranean Diet In Healthy Aging. J. Nutr. Health Aging.

[30] Margină D., Ungurianu A., Purdel C., Nițulescu G.M., Tsoukalas D., Sarandi E., Thanasoula M., Burykina T.I., Tekos F., Buha A. (2020). Analysis of the Intricate Effects of Polyunsaturated Fatty Acids and Polyphenols on Inflammatory Pathways in Health and Disease. Food Chem. Toxicol..

[31] Petrella C., di Certo M.G., Gabanella F., Barbato C., Ceci F.M., Greco A., Ralli M., Polimeni A., Angeloni A., Severini C. (2021). Mediterranean Diet, Brain and Muscle: Olive Polyphenols and Resveratrol Protection in Neurodegenerative and Neuromuscular Disorders. Curr. Med. Chem..

[32] Kent G., Kehoe L., Flynn A., Walton J. (2022). Plant-Based Diets: A Review of the Definitions and Nutritional Role in the Adult Diet. Proc. Nutr. Soc..

[33] Bach-Faig A., Berry E.M., Lairon D., Reguant J., Trichopoulou A., Dernini S., Medina F.X., Battino M., Belahsen R., Miranda G. (2011). Mediterranean Diet Pyramid Today. Science and Cultural Updates. Public Health Nutr..

[34] Gao X., Chen H., Fung T.T., Logroscino G., Schwarzschild M.A., Hu F.B., Ascherio A. (2007). Prospective Study of Dietary Pattern and Risk of Parkinson Disease. Am. J. Clin. Nutr..

[35] Yin W., Löf M., Pedersen N.L., Sandin S., Fang F. (2021). Mediterranean Dietary Pattern at Middle Age and Risk of Parkinson’s Disease: A Swedish Cohort Study. Mov. Disord..

[36] Morris M.C., Tangney C.C., Wang Y., Sacks F.M., Barnes L.L., Bennett D.A., Aggarwal N.T. (2015). MIND Diet Slows Cognitive Decline with Aging. Alzheimer’s Dement..

[37] Agarwal P., Wang Y., Buchman A.S., Holland T.M., Bennett D.A., Morris M.C. (2018). MIND Diet Associated with Reduced Incidence and Delayed Progression of ParkinsonismA in Old Age. J. Nutr. Health Aging.

[38] Alcalay R.N., Gu Y., Mejia-Santana H., Cote L., Marder K.S., Scarmeas N. (2012). The Association between Mediterranean Diet Adherence and Parkinson’s Disease. Mov. Disord..

[39] Mischley L.K., Lau R.C., Bennett R.D. (2017). Role of Diet and Nutritional Supplements in Parkinson’s Disease Progression. Oxid. Med. Cell. Longev..

[40] Paknahad Z., Sheklabadi E., Derakhshan Y., Bagherniya M., Chitsaz A. (2020). The Effect of the Mediterranean Diet on Cognitive Function in Patients with Parkinson’s Disease: A Randomized Clinical Controlled Trial. Complement. Ther. Med..

[41] Paknahad Z., Sheklabadi E., Moravejolahkami A.R., Chitsaz A., Hassanzadeh A. (2022). The Effects of Mediterranean Diet on Severity of Disease and Serum Total Antioxidant Capacity (TAC) in Patients with Parkinson’s Disease: A Single Center, Randomized Controlled Trial. Nutr. Neurosci..

[42] Maraki M.I., Yannakoulia M., Stamelou M., Stefanis L., Xiromerisiou G., Kosmidis M.H., Dardiotis E., Hadjigeorgiou G.M., Sakka P., Anastasiou C.A. (2019). Mediterranean Diet Adherence Is Related to Reduced Probability of Prodromal Parkinson’s Disease. Mov. Disord..

[43] Molsberry S., Bjornevik K., Hughes K.C., Healy B., Schwarzschild M., Ascherio A. (2020). Diet Pattern and Prodromal Features of Parkinson Disease. Neurology.

[44] Rusch C., Beke M., Tucciarone L., Nieves C., Ukhanova M., Tagliamonte M.S., Mai V., Suh J.H., Wang Y., Chiu S. (2021). Mediterranean Diet Adherence in People with Parkinson’s Disease Reduces Constipation Symptoms and Changes Fecal Microbiota After a 5-Week Single-Arm Pilot Study. Front. Neurol..

[45] McLaren D.S. (1997). Coronary Heart Disease in Seven Countries. 1970. Nutrition.

[46] Parkinson L., Cicerale S. (2016). The Health Benefiting Mechanisms of Virgin Olive Oil Phenolic Compounds. Molecules.

[47] Hughes K.C., Gao X., Kim I.Y., Rimm E.B., Wang M., Weisskopf M.G., Schwarzschild M.A., Ascherio A. (2016). Intake of Antioxidant Vitamins and Risk of Parkinson’s Disease. Mov. Disord..

[48] Bianchi V.E., Herrera P.F., Laura R. (2021). Effect of Nutrition on Neurodegenerative Diseases. A Systematic Review. Nutr. Neurosci..

[49] Hornedo-Ortega R., Cerezo A.B., de Pablos R.M., Krisa S., Richard T., García-Parrilla M.C., Troncoso A.M. (2018). Phenolic Compounds Characteristic of the Mediterranean Diet in Mitigating Microglia-Mediated Neuroinflammation. Front. Cell. Neurosci..

[50] Angeloni C., Malaguti M., Barbalace M.C., Hrelia S. (2017). Bioactivity of Olive Oil Phenols in Neuroprotection. Int. J. Mol. Sci..

[51] Serreli G., Deiana M. (2020). Extra Virgin Olive Oil Polyphenols: Modulation of Cellular Pathways Related to Oxidant Species and Inflammation in Aging. Cells.

[52] Bucciantini M., Leri M., Nardiello P., Casamenti F., Stefani M. (2021). Olive Polyphenols: Antioxidant and Anti-Inflammatory Properties. Antioxidants.

[53] Nagpal R., Mainali R., Ahmadi S., Wang S., Singh R., Kavanagh K., Kitzman D.W., Kushugulova A., Marotta F., Yadav H. (2018). Gut Microbiome and Aging: Physiological and Mechanistic Insights. Nutr Healthy Aging.

[54] Yu D., Meng X., de Vos W.M., Wu H., Fang X., Maiti A.K. (2021). Implications of Gut Microbiota in Complex Human Diseases. Int J Mol Sci.

[55] de Filippis F., Pellegrini N., Vannini L., Jeffery I.B., la Storia A., Laghi L., I Serrazanetti D., di Cagno R., Ferrocino I., Lazzi C. (2016). High-Level Adherence to a Mediterranean Diet Beneficially Impacts the Gut Microbiota and Associated Metabolome. Gut.

[56] Gutiérrez-Díaz I., Fernández-Navarro T., Sánchez B., Margolles A., González S. (2016). Mediterranean Diet and Faecal Microbiota: A Transversal Study. Food Funct..

[57] Garcia-Mantrana I., Selma-Royo M., Alcantara C., Collado M.C. (2018). Shifts on Gut Microbiota Associated to Mediterranean Diet Adherence and Specific Dietary Intakes on General Adult Population. Front. Microbiol..

[58] Tomova A., Bukovsky I., Rembert E., Yonas W., Alwarith J., Barnard N.D., Kahleova H. (2019). The Effects of Vegetarian and Vegan Diets on Gut Microbiota. Front. Nutr..

